# Fortification of Maize Tortilla with an Optimized Chickpea Hydrolysate and Its Effect on DPPIV Inhibition Capacity and Physicochemical Characteristics

**DOI:** 10.3390/foods10081835

**Published:** 2021-08-09

**Authors:** Karla A. Acevedo-Martinez, Elvira Gonzalez de Mejia

**Affiliations:** Department of Food Science and Human Nutrition, University of Illinois at Urbana-Champaign, Urbana, IL 61801, USA; karlaaa2@illinois.edu

**Keywords:** chickpea, enzymatic hydrolysis, hydrolysate, DPPIV, bromelain, bioactive peptides, type 2 diabetes, protein fortification

## Abstract

Chickpea hydrolysates have shown bioactivity towards type 2 diabetes by inhibiting dipeptidyl peptidase (DPPIV) activity. The objective was to compare the effect of adding different levels of an optimized bromelain hydrolysate from chickpea isolated protein on DPPIV inhibition capacity and physicochemical properties of maize tortilla. White and blue maize tortillas, with no added chickpea hydrolysates were compared with fortified tortillas at the levels of 5%, 10%, and 15% *w*/*w*. Changes in color (L* a* b*, hue angle, and ΔE), texture (hardness, cohesiveness, and puncture force), and moisture were tested. Soluble protein determination and SDS-PAGE electrophoresis were used to characterize the protein profiles, and LC-MS-MS was used to sequence the peptides. DPPIV inhibition was evaluated before and after simulated gastrointestinal digestion. Peptides in the hydrolysates had high hydrophobicity (7.97–27.05 kcal * mol ^−1^) and pI (5.18–11.13). Molecular docking of peptides showed interaction with DPPIV with an energy of affinity of –5.8 kcal/mol for FDLPAL in comparison with vildagliptin (−6.2 kcal/mol). The lowest fortification level increased soluble protein in 105% (8 g/100 g tortilla). DPPIV inhibition of white maize tortilla increased from 11% (fresh control) to 91% (15% fortification), and for blue tortilla from 26% to 95%. After simulated digestion, there was not a difference between blue or maize tortillas for DPPIV inhibition. Fortification of maize tortilla with chickpea hydrolysate inhibits DPPIV and can potentially be used in the prevention and management of type 2 diabetes. However, due to observed physicochemical changes of the fortified tortilla, sensory properties and consumer acceptance need to be evaluated.

## 1. Introduction

Chickpea (*Cicer arietinum* L.) is one of the most consumed pulses worldwide. It has shown important bioactivities such as antioxidant capacity; antifungal, antibacterial, and analgesic properties; and angiotensin I-converting enzyme inhibition; as well as hypocholesterolemic, anticancer, and anti-inflammatory properties [1,2,3,4]. Other studies have reported antidiabetic properties of chickpea, related to its content of protein and phenolic compounds [2,5,6,7,8].

Diabetes mellitus (DM) in the United States (U.S.) affects 10.5–13.0% of the population and represents the seventh leading cause of death [9]. DM is associated with heart disease and stroke, blindness, kidney failure, and lower-limb amputation [10]; thus, DM is a burden on public health. Effective management mechanisms are needed to decrease the burden of DM; an exercise program and a diet that includes pulses are effective in managing the disease [11].

The use of food ingredients that provide health benefits as well as plant protein alternatives have been gaining popularity among consumers. For example, 65% of people consider functional benefits when looking for food options [12]. Mintel [13] reported an increasing trend in the U.S. to include plant-based foods in daily diets without sacrificing taste. A common way to include plant-based options in the diet is by using legumes and pulses. Mintel [13] reported that in the U.S., 81% of consumers chose beans, chickpeas, or fava beans.

On the other hand, globally, maize is the most important grain in terms of production. During the period 2018–2019, the global production of maize was 1.09 billion metric tons, with the U.S. being the biggest producer (345 million metric tons), followed by China, Brazil, the European Union, and Argentina [14]. Maize is an essential food for many countries in Latin America, not only because of its social and cultural relevance but also for its economic impact [15]. Maize is indispensable for the production of tortillas, which in 2017 represented in Mexico an income of USD 2.1 million [16]. In the U.S., the tortilla and corn-based snacks industry has been increasing steadily. In this country, the projected sales for tortillas are projected to be approximately USD 3.659 million by 2024 [17]. Maize is deficient in lysine and tryptophan, and its fortification improves the content of protein, fiber, and bioactive compounds with antioxidant capacity [18,19,20,21].

Traditionally, tortillas are made from nixtamalized maize masa or from reconstituted nixtamalized maize flour. Nixtamalization is an alkaline process of maize that consists in soaking or cooking the grains in water and adding 1–2% of Ca(OH)_2_ per kilogram of maize [22,23]. This process changes the structure and nutritional value of maize by removing the pericarp, causing partial gelatinization of starches, changing protein solubility, releasing niacin, and increasing calcium content by up to 400 times [24,25,26]. As a result, the rheological, functional, and textural properties of the masa and final product change, influencing its acceptability, especially the improved workability into a dough.

Some maize varieties are commonly used to prepare tortillas due to their physical characteristics, such as color and texture [21]. Particularly blue and purple maize have been studied for their anti-inflammatory, antiadipogenic, and antidiabetic properties related to their content of anthocyanins [27]. One goal for the control of DM is to inhibit enzymes that metabolize carbohydrates such as α-amylase and α-glucosidase and to inhibit dipeptidyl peptidase (DPPIV), increase incretin, prolong postprandial insulin action, and inhibit glucagon release [28,29].

DPPIV is a glycoprotein that cleaves N-terminal dipeptides from the penultimate position of cytokines, growth factors, neuropeptides, and the incretin hormones [28]. Although having different actions in the substrates mentioned, the central relevance related to type 2 diabetes (T2D) is that DPPIV interacts with incretins. Incretin hormones potentiate insulin secretion and are secreted from the gut within minutes after food intake; they stimulate insulin secretion and suppress glucagon release depending on the blood glucose level. Therefore, by inhibiting DPPIV, its interaction with incretins is reduced, leading to greater bioavailability of incretins and thus prolonging the half-life of insulin action [28].

Tortillas are an ideal medium to investigate protein fortification given their wide popularity in the United States, where their market value approximates USD 12,324.4 million, yet their protein quality is low [30]. Acevedo Martinez and Gonzalez de Mejia [31] produced an optimized chickpea hydrolysate (CPH) capable of high DPPIV inhibition. Therefore, we hypothesized that fortification of maize tortillas with increasing levels of CPH would increase soluble protein concentration and DPPIV inhibition capability. The objective was to compare the effect of adding different levels of an optimized bromelain hydrolysate from chickpea isolated protein on DPPIV inhibition capacity and the physicochemical properties of white and blue maize tortillas.

## 2. Materials and Methods

### 2.1. Materials

The kabuli Sierra chickpea seeds were purchased from Palouse Brand in southeastern Washington State (WA, USA). Chickpeas were seeded on 9 May 2019 and harvested by 12 October 2019. The dry grains were stored in cloth bags at 4 °C until use. White single-origin heirloom nixtamalized maize (white olotillo) flour sourced from the tropical (coastal) climes of Oaxaca, Mexico, was purchased from Masienda (Los Angeles, CA, USA). The heirloom blue cónico nixtamalized maize flour grown in the highlands of Atlacomulco in Estado de México was purchased from Masienda (Los Angeles, CA, USA). Based on the information taken from the label of the package, both white and blue flours had a total protein concentration of 10%, and were used for the tortilla preparation. Enzymes, human DPPIV (EC 3.4.14.5), pancreatin from porcine pancreas (EC 232.468.9), pepsin (EC 3.4.23.1), and bromelain from pineapple stem (EC 3.4.22.32) were purchased from Sigma-Aldrich (St. Louis, MO, USA). DPPIV-GLO^®^ protease assay kit was purchased from Promega (Madison, WI, USA). Other reagents were obtained from Sigma-Aldrich.

Bromelain is a cysteine endopeptidase with broad specificity towards peptide bonds, and it was chosen to represent a more affordable protease option [32]. Pepsin is also a peptidase, an aspartic endopeptidase with broad specificity that cleaves to tyrosine, phenylalanine, and tryptophan. Pancreatin, being a combination of digestive enzymes (amylase, trypsin, lipase, ribonuclease, and protease), also has a broad spectrum that hydrolyzes proteins, starch, and fats [33].

### 2.2. Chickpea Protein Isolation and Optimized Enzymatic Hydrolysis

Dry chickpeas were precooked in boiling water at 98 °C for 15 min and were placed on ice to stop the cooking process. Precooked chickpeas were blended into a slurry in a 1:20 chickpea-to-water ratio until a smooth homogenous texture was obtained. The heat- processed chickpea slurry was filtered using a cheesecloth, centrifuged to separate the solids, and processed the same day to extract the protein. The extraction of chickpea protein was conducted by the method of Sánchez-Vioque et al. [34], with some adaptations. Chickpea protein was isolated based on the isoelectric point (4.5). The pH was first adjusted to 9.0 using 1 M NaOH, followed by continuous stirring at 300 rpm for 1 h at 24–25 °C. The chickpea suspension was then centrifuged at 5000× *g* for 20 min at 4 °C and the solid was discarded. The proteins in the supernatant were then precipitated by adding 1 M HCl until a pH 4.5 was reached, followed by another centrifugation at 5000× *g* for 20 min at 4 °C. The supernatant was disposed of, and the pellet of the protein isolates was freeze-dried in a Freeze Dryer 4.5 (Labconco, Kansas, MO, USA) and stored at −20 °C until hydrolysis.

Optimized enzymatic hydrolysis conditions were used, following the procedure described by Acevedo Martinez and Gonzalez de Mejia [31]. The enzymatic hydrolysis was carried out using 400 mg of chickpea protein isolate and 40 mg of bromelain (≥3 units/mg protein) (1:10 enzyme-to-substrate ratio) in continuous stirring for 60 min. The temperature was set constant at 25 °C, and pH was at 4.5. Enzymes were then inactivated in a water bath at 79 °C for 10 min, centrifuged to separate solids, and filtered through a 0.22 μm cutoff syringe. Hydrolysates were freeze-dried and stored at −20 °C.

### 2.3. LC-ESI-MSMS Peptide Sequencing

Hydrolysates of the chickpea protein isolates (2 mg/mL protein) were centrifuged in a Fisher Scientific Mini Centrifuge (6000× *g*) for 10 min, and the supernatant was then filtered through 0.45 µm. The peptides were analyzed by LC-ESI-MSMS using the SYNAPTG2-S MS mass spectrometer (Waters, Milford, MA, USA), equipped with a Waters Acquity UPLC HSS T3 1.8 um 2.1 × 100 mm (−0.2 to 0 °C). Separation of the components was performed by using a mobile phase of solvent A (95% H_2_O, 5% acetonitrile (ACN), and 0.1% formic acid) and solvent B (95% ACN, 5% H_2_O, and 0.1% formic acid) using a flow rate of 200 μL/min. The elution was in a linear gradient (0 min, 90% A; 2 min, 90% A; 40 min, 65% A; 60 min, 10% A; 65 min, 10% A; 66 min, 90% A; 80 min, 90% A). Analysis was set at a flow rate of 0.400 mL/min for a total time of 15 min. Temperature during the analysis was 6.83 °C, with an average pressure of 10885.0 psi. The data were analyzed using the MassLynx V4.1 software (Waters Corporation, Milford MA, USA). Chromatogram peaks with at least 50% intensity were selected and analyzed. A mass spectrum was generated for each peak of the chromatogram, and only the most abundant peptide fragments (%intensity > 50%) were selected for sequencing. The sequence of amino acids was identified based on accurate mass measurements and tandem MS fragmentation using the MassBank database, and the amino acids were presented in one letter nomenclature. The parental protein was identified with BLAST tool (https://blast.ncbi.nlm.nih.gov/Blast.cgi (accessed on 23 March 2021)). Peptides’ structures and properties were predicted using PepDraw (Thomas C. Freeman, 2015) tool, and the potential bioactivity of the peptides was predicted using the BIOPEP database.

### 2.4. Molecular Docking

Autodock Vina (Scripps Research, La Jolla, CA, USA) was used for the analysis [8]. The structure of the predicted sequences was drawn using MarvinSketch (ChemAxon, Boston, MA, USA). Crystallographic structure of DPP-IV (PDB ID: 6B1E) was retrieved from the Protein Data Base and was used to evaluate the anti-diabetic potential of individual sequences found in the hydrolysates [35]. The docking position was determined using the drugs previously docked onto the abovementioned crystallographic system. Using Discovery Studio V4.1, the ligands and water molecules were removed from the original template. Using Autodock Tools, the ligand was inputted into the template based on the active site of previously docked anti-diabetic drugs [36]. The energy of affinity with the active site of the enzyme was calculated using Autodock Vina [37]. Images of this interaction, which outlines the amino acids in the peptide sequence and in the enzyme participating in the interaction, were generated using Discovery Studio V4.1.

### 2.5. Tortilla Preparation and Fortification with Chickpea Hydrolysate

An alkaline-treated (nixtamalized) maize tortilla formulation was produced and added with four different optimized chickpea protein hydrolysates ratios: 0, 5, 10, and 15% *w*/*w* (hydrolysate weight/ tortilla weight). For one tortilla, six grams of maize flour (blue or white) were manually mixed with 12 mL of water until the masa was formed, after which it was stored in plastic bags at 4 °C for 24 h before the cooking process. The fortification treatments, 0.500 g (5%), 1 g (10%), or 1.5 g (15%) of CPH were fully solubilized in the 12 mL water before the addition to the maize flour. The weight of chickpea hydrolysate added was replaced from the original 6 g of flour to keep the total weight of masa a constant 6 g dry material and 12 mL water per tortilla; an equivalent part of flour was removed to keep the final tortilla weight constant. After 24 h, the masa was mixed again and weighted as 13 g masa balls (“testales”). Using a mechanical tortilla press, each ball was pressed four times, rotating the masa 90° clockwise in between each press to have a homogeneous thickness. Each tortilla was cooked on a flat top at 232 °C, flipping it each 20 s for 2 min. Once they were cooked, each 10 g tortilla was put on a rack to cool down for 20 min before performing physical analysis. Lastly, tortillas were stored individually in polyethylene Ziplock bags at 4 °C for further analysis.

Fresh and stored fortified tortillas (48 h and 7 days) were analyzed to determine texture, moisture, color, soluble protein, and DPPIV inhibition.

### 2.6. Soluble Protein Determination (BCA)

Chickpea soluble protein was determined by the bicinchoninic acid (BCA) procedure [38]. In summary, bovine serum albumin (BSA) was diluted with tris buffered saline (TBS). TBS was used as standard. Tortilla controls and fortified were tested using 1 g of sample crushed with mortar and suspended with water in a 1:10 ratio. After centrifuging the suspension to separate the insoluble solids and filtering through a 0.22 μm cutoff syringe, a further 1:10 dilution with TBS was prepared, and after addition of BCA reagent, they were analyzed in a clear 96-well plate and absorbance was read at 690 nm. A standard curve and the following equation were used in the calculation as follows:

(1) $$y=0.0002x-0.0075\;\;R^{2}=0.997$$

### 2.7. SDS PAGE Electrophoresis

SDS-PAGE was carried out using the technique reported by Laemlli [39]. Filtered tortilla aliquots were mixed in 1 mL sample buffer (0.5 M Tri-HCl pH 6.8, glycerol, 10% sodium dodecyl sulfate (SDS), 1% bromophenol blue, and β-mercaptoethanol) heated at 98 °C for 5 min. Then, 25 µg of protein was applied to the gel wells of 16.5% Tris-Tricine Mini Protean Precast gels (Bio-Rad, Hercules, CA, USA). Gels were stained with Simply Blue solution for 1 h and then washed overnight with distilled water. Images were acquired in the ImageQuant LAS4000 (GE Healthcare, Uppsala, Sweden). Proteins were compared with the Spectra Multicolor Low Range Protein Ladder (Thermo Fisher, Waltham, MA, USA) (1.7 kDa–40 kDa).

### 2.8. Color Analysis

Color was measured by a colorimeter (Hunter LabScan XE, USA) in terms of L* (lightness), a* (degree of redness to greenness), and b* (degree of yellowness to blueness). Tortillas controls and after fortification were measured at three points on the tortilla and averages were reported. The values ∆*E* compared with the control were calculated using the equation:

(2) $$\Delta E=\sqrt{{\left(L_{2}^{*}-L_{1}^{*}\right)}^{2}+{\left(a_{2}^{*}-a_{1}^{*}\right)}^{2}+{\left(b_{2}^{*}-b_{1}^{*}\right)}^{2}}$$

Hue angle was calculated by:

(3) $$h=tan^{-1}\left(\frac{a^{*}}{b^{*}}\right)$$

### 2.9. Moisture Analysis

The moisture analysis was performed in fresh tortillas as well as after 48 h and after 7 days of storage in polyethylene Ziplock bags. A Halogen Moisture Analyzer HR83 was used to test 1 g of tortillas, and moisture content was determined as ([initial mass − dried mass]/initial mass) × 100.

### 2.10. Texture Analysis: Hardness, Cohesiveness, and Penetration Test

To analyze the changes in the tortillas due to the addition of CPH, texture profile analysis (TPA; analysis of hardness, cohesiveness, and puncture force) was conducted using a TA-XT2i Texture Analyzer (Texture Technologies Corp., Scarsdale, NY, USA). For TPA, tortilla circular portions of controls or treatments (7.2 cm diameter and 1.75 ± 0.15 mm thickness) were cut from the center of the tortilla with a mold after being tempered at room temperature (21 °C). They were compressed between a metallic TA 90 plate and a TA 25 aluminum probe of 2” diameter. Pretest speed and posttest speed were 3 mm/s, test speed was 1 mm/s with a target mode strain of 60% and 3 s between bites. Trigger type force was set at 10 g. Puncture test was carried out with a cylinder penetrometer probe (4 mm); the probe was passed through the tortilla sample. Test parameters were set at 5 mm/s of pre-speed and post-speed, 2 mm/s of test speed, and 20 g of trigger. Tortillas from each fortification treatment were evaluated after 20 min, 48 h, and 7 days stored at 4  °C. The means from three replicates are reported.

### 2.11. Dipeptidyl Peptidase Inhibition Biochemical Assay

The potential of DPPIV inhibition was tested in the composite chickpea hydrolysate and in fresh tortillas before and after SGID. DPPIV inhibition was measured using the DPP-IVGLO^®^ Protease Assay (G8351, Promega, Madison, WI, USA). A 25 μL of DPP-IVGLO^®^ reagent was added to a white-walled 96-well plate containing either 20 μL of water as blank, 20 μL sitagliptin (control) solution (0.1 μM), or 20 μL of white and blue fortified tortilla aliquots. An enzyme control was used adding 20 μL of water and 5 μL of purified DPPIV human enzyme (10 ng/mL). Additionally, 25 μL of DPP-IVGLO^®^ reagent was added to every well. Luminescence was measured after mixing and incubating for 30 min using a Synergy2 multiwell plate reader (Biotek Instruments, Winooski, VT, USA). Percentage inhibition was calculated from the blank and enzyme control for each chickpea hydrolysate analyzed.

### 2.12. Simulated Gastrointestinal Digestion (SGID) of Chickpea-Fortified Tortillas

To evaluate the effect of fortification on tortilla digestibility, a simulated digestion method was performed following the methodology used by Megías et al. [40]. In detail, one g of tortilla was used to test controls or treatments. The tortillas were crushed with a mortar and washed with water in a 1:10 ratio. An enzyme/substrate (E/S) ratio of 1:20 was used for both pepsin (5337.5 U/mL) and pancreatin (8 × USP). For pepsin digestion, the pH was adjusted to 2.0, and the chickpea isolate dilution was brought to 37 °C, the enzyme was added, and the solution was stirred for 1.5 h. Then, pH was adjusted to 6.8 and pancreatin was added and stirred for 1.5 h. Enzymes were inactivated in a water bath at 79 °C for 10 min. Digests were centrifuged to separate solids at 20,000× *g* for 5 min at 4 °C. Aliquots were filtered through a 0.22 μm cutoff syringe filter and frozen at −20 °C until further analysis.

### 2.13. Statistical Analysis

Independent triplicates were tested and analyzed. Data are expressed as mean ± standard deviation. Statistical analyses and differences among groups were tested according to their statistical significance using one-way ANOVA and Tukey’s test. Differences were considered significant at *p* < 0.05. Correlation analysis was performed to evaluate associations among variables.

## 3. Results

### 3.1. Peptide Sequencing, Soluble Protein, and DPPIV Inhibition

**Figure 1:**
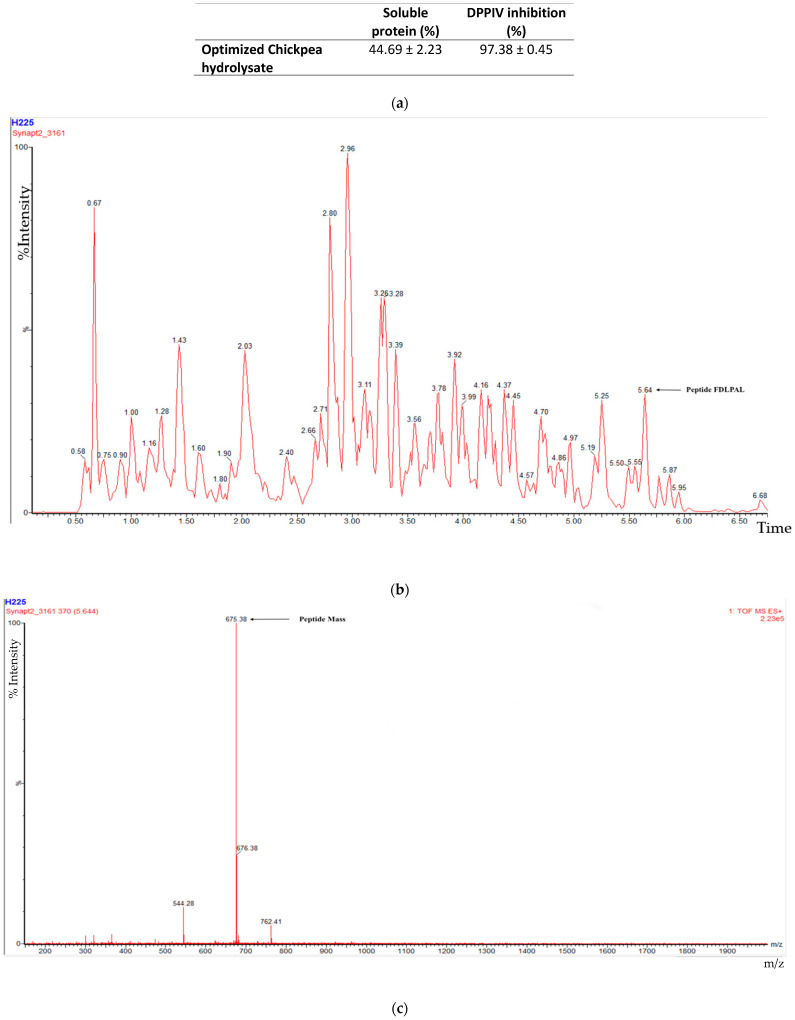
(**a**) Soluble protein and DDPIV inhibition of composite sample (chickpea-optimized hydrolysate). (**b**) Mass spectra indicating elution time. (**c**) Spectra showing peptide mass, and precursor ion *M/z* value of peptide FDLPAL.

To analyze the initial characteristics of the composite chickpea hydrolysate produced, soluble protein and DPPIV inhibition were determined (Figure 1a). Information on the peptides originated from chickpea storage proteins is shown in Figure 1b,c (FDLPAL), where the peptide mass and precursor ion for this peptide is shown. In addition, further analysis of another peptide sequence from lectin is shown in Figure S1.

Table 1 shows the peptide sequences found in the composite chickpea hydrolysate used to fortify the tortillas. All peptides included in Table 1 were found to have potential to inhibit DPPIV using the BIOPEP database. The characteristics of the peptides include a wide range of pI (3.12–11.13) and hydrophobicity (7.21–27.05 kcal/mol). These features indicate a wide potential application of the hydrolysate since it is soluble at pH values from 2 to 12.

**Figure 2:**
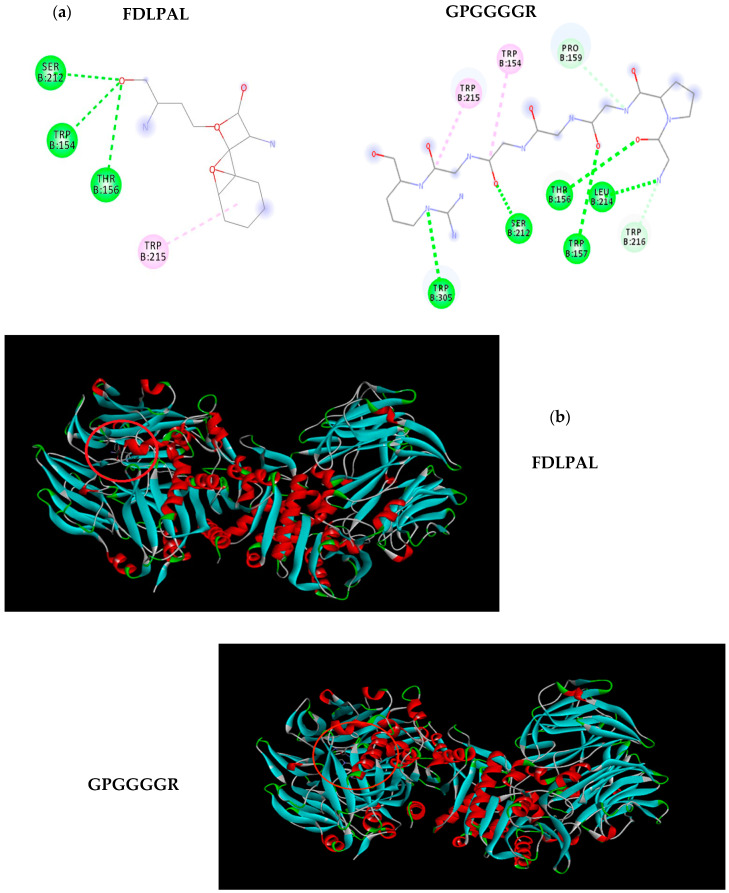
Docking interaction and DPPIV enzyme–peptide location for optimized chickpea bromelain hydrolysate peptide sequences FDLPAL and GPGGGGR. (**a**) FDLPAL, best pose of chickpea peptide Phe-Asp-Leu-Pro-Ala-Leu (structure in gray, left) in the molecular docking study of sequences with DPPIV. GPGGGGR, best pose of chickpea peptide Gly-Pro-Gly-Gly-Gly-Gly-Arg (structure in gray, right) in the molecular docking study of sequences with DPPIV. (**b**) Position of peptide Phe-Asp-Leu-Pro-Ala-Leu (circled in red, left) in the molecular docking study with DPPIV. Position of peptide Gly-Pro-Gly-Gly-Gly-Gly-Arg (circled in red, right) in the molecular docking study with DPPIV.

The analysis of molecular docking of peptides (Figure 2) shows interactions with the crystal structure of DPPIV with an energy of affinity of −5.8 kcal/mol for FDLPAL with the amino acids SER 212, TRP 154, THR 156, and TRP 215. The energy of affinity for the sequence GPGGGGR was −5.7 kcal/mol interacting with TRP154, PRO 159, TRP 305, TRP 157, LEU 214, and TRP 216 in addition to the amino acids TRP215, SER 212, and THR 156, similar to peptide FDLPAL. Energy of affinity was compared with that of vildagliptin of −6.2 kcal/mol. Vildagliptin (LAF237) is an orally active antihyperglycemic agent that selectively inhibits DPPIV.

SDS-PAGE profile and soluble protein (Figure S2 and Table 2) show the positive impact of the addition of the hydrolysates to the tortillas. The soluble protein of the composite CPH was 44.69 ± 2.23%. All CPH fortification ratios showed significant difference (*p* < 0.05) in soluble protein from the control and among them, but there was no difference between maize types for the same fortification levels.

Changes in color (Table 2) were observed in both blue and white maize tortillas. Most color changes were observed in blue maize tortillas (*p* < 0.05). In contrast, 5% fortification of white maize tortillas did not show significant changes in either L*, a*, or b* values. Differences in ∆*E* can be interpreted as changes perceived by the human eye in comparison with the control. It can be due to the light yellowish color of the hydrolysate when solubilized in water. Losses in anthocyanins due to cooking and changes in pH in blue maize masa and tortillas have been studied previously [41]. In the present study, cooking time and temperature conditions were the same for all the tortillas, including the control.

### 3.2. Moisture Analysis

The presence of CPH in the fortified tortilla slightly affected its moisture content (Table 3). In fresh tortillas, for both blue and white maize tortilla, moisture content was higher for the 10% fortification ratio compared with the control tortillas. The second highest moisture content was for the 5% fortification ratio in white tortillas, while 15% and 0% were not significantly different. In fresh blue tortillas, there was no difference in moisture content at 0%, 5%, or 15% fortification ratios. At 48 h of storage, 0% fortification had the highest moisture content in white tortillas, while blue tortillas did not show a difference among fortification ratios. After 7 days of storage, the trend was similar, with no significant change in moisture content for both blue and white tortillas. However, the presence of the hydrolysate from 5% to 15% allowed the tortillas, blue and white, to remain with constant moisture content over storage time as when fresh—different from tortillas with no hydrolysate (white and blue), which had a significant moisture increase after 48 h. For white and blue tortillas, this increase remained constant even after 7 days, and it was statistically similar to the 48 h data (*p* > 0.05).

In this study, the tortillas weighted 10 g and had an average thickness of 1.95 ± 0.14 mm for white tortillas and 2.04 ± 0.10 for blue maize tortillas, with no difference among fortification ratios. The average diameter (Table 3) for white maize tortillas was of 81.06 ± 1.33 mm, with no difference among fortification ratios. For blue maize tortillas of 0–10% fortification level, the average diameter was 80.69 ± 1.78, and for the 15%, the fortification ratio was 78 ± 0.94 mm.

### 3.3. Texture Analysis

Texture analysis showed that when tortillas were fresh, there was a relationship between fortification levels and hardness (Table 4). For fresh tortillas, white maize treatments resulted in higher hardness values at 10% and 15% fortification ratios (27.47 and 25.58 N, respectively), in comparison with the control (13.56 ± 1.66, *p* < 0.05). For fresh blue maize tortillas, only the 15% fortification showed significant lower hardness values (16.71 ± 2.63 N). After 7 days of storage, the hardness of the blue tortillas at 10% fortification level was 21.53 N, higher than any other fortification level (*p* < 0.05). However, when compared with the initial hardness (fresh tortilla), 10% and 15% fortification levels did not show a difference (*p* > 0.05) in this texture parameter. White tortillas with 10% fortification ratio showed the highest hardness value (32.38 ± 1.12 N), followed by the 15% fortification ratio (25.39 ± 1.51 N) (*p* < 0.05). After 7 days, tortillas with no hydrolysate had an air pocket, which separated the tortillas into two layers. They also felt soggier and blander than the fortified tortillas that did not have air pockets. TPA graphical representation of fresh and after 7 days analysis for the control and for 15% fortification are shown in Figure S3. Values of cohesiveness and penetration force did not show significant trends with increasing fortification.

### 3.4. DPPIV Inhibitory Activity

**Figure 3:**
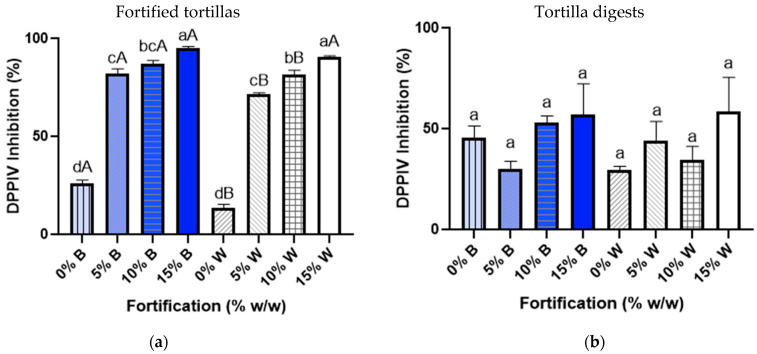
DPPIV inhibition of white and blue tortillas fortified at 0, 5, 10, and 15% with optimized chickpea hydrolysate with DPPIV activity obtained from isolated protein using bromelain. (**a**) Comparison among fortification ratios either for white or blue maize is represented with lowercase letters (*p* < 0.05). Comparison between white and blue maize for specific fortification levels is indicated with capital letters (*p* < 0.05). (**b**) DPPIV activity after a simulated gastrointestinal digestion (SGID) using pepsin and pancreatin.

The DPPIV inhibitory activity of the composite sample of CPH was of 97.38 ± 0.45%. The statistical analysis of DPPIV was performed among fortification ratios, either for white or blue maize tortillas (Figure 3a, lowercase letters), and then between white and blue maize tortillas for specific fortification levels (Figure 3a, capital letters). The results show significant differences (*p* < 0.05) between white and blue maize at 0%, 5%, and 10% fortification levels, with blue maize tortillas having higher DPPIV inhibition in comparison with the control: 82% inhibition at 5% fortification level and 87% inhibition at 10% fortification level. At 15% fortification, there were not significant differences between maize varieties (95% for blue maize tortilla and 91% for white maize tortilla). White maize tortilla increased from 11% (0% fortification) to 72%, 82%, and 91% of DPPIV inhibition activity as the fortification level increased. In order to assure that the observed DPPIV activity remained after digestion, a SGID experiment using pepsin and pancreatin was performed. After SGID, there was no difference (*p* < 0.05) between blue or maize tortillas for DPPIV inhibition, showing a range from 41% to 61% DPPIV inhibition (Figure 3b).

## 4. Discussion

Analyzing the solubility in the composite chickpea hydrolysate, we observed similar behavior reported in previous studies [42] as a result of the enzymatic hydrolysis that provides better solubility, stability, and functional properties to the hydrolysates in comparison with protein concentrates or isolates. The increased solubility in protein hydrolysates is explained by the reduced molecular weight and increased hydrophilicity resulting from the increase in free carboxyl and amine groups [43]. Peptide sequence FDLPAL has been previously reported [31] as originating from storage proteins. One more sequence found that GPGGGGR originated from trypsin inhibitors, showing the resistance of these antinutrients to hydrolysis conditions and heat treatments.

Peptide fraction LL from the sequence LLR reported in the present study was predicted previously [4] from a theoretical hydrolysis of chickpea legumin by stem bromelain with other bioactivity in addition to DPPIV inhibition, such as glucose uptake stimulator. Other sequences reported from bromelain hydrolysis [8] found peptides with a repeated presence of glycine (GGG and GGGG) and glycine alanine and arginine (GAR, GR), which were also common in the sequences found in this study. Findings can be related to the unspecific activity of the bromelain to hydrolyze proteins. Bromelain, a cysteine endopeptidase has a preference for peptide bonds distant from the N- or C-termini [44]. Due to this characteristic, we were able to observe a wide range of molecular weights in the peptides identified (400.27–1385.66 g/mol). In other studies [8,31], similar findings were reported. These researchers identified peptide sequences as large as 1670.8 g/mol and as small as 549.29 g/mol. Lastly, peptide sequence KEGGGTGTGAAR identified in previous in silico analysis from pepsin–pancreatin hydrolysis [4] reported similar peptide characteristics in pI, charge, and hydrophobicity as the KDGGTAPAAGSGGGGAR sequence that was found in the studied composite chickpea hydrolysate.

The initial soluble protein at 0% tortilla fortification was of 4.0 g of soluble protein per 100 g of tortilla, lower than the total protein reported in the maize flour packaging (10 g of total protein per 100 g of flour). A similar phenomenon has been reported, and it has been related to the aggregation of the proteins during masa making, due to mixing and degradation of globulins during the cooking process [45]. However, fortifications at the 5% level showed an increase (*p* < 0.05), compared with the tortilla with no fortification, in soluble protein concentration of 105% (8 g/100 g tortilla) for both blue and white tortilla and up to 13 g/100 g tortilla at a 15% fortification ratio. It can be observed in the SDS-PAGE gels (Figure S2) that the hydrolysate profile predominates even after the cooking process. The >10 kDa protein bands from the maize flour correspond to zein protein [46]; other proteins may have degraded by the processing conditions. The remaining proteins present in the tortillas are of lower molecular weight (<10 kDa). The intensity of the bands were observed to increase as the fortification ratios increased (Figure S2).

According to Food and Drug Administration (FDA) regulations, food fortification is considered when the fortified food contains at least 10% or more of the daily reference value for protein expressed as a percentage of the daily value per 100 g of food. The daily reference value for protein is 50 g per day based on a 2000 calorie daily diet [47]; therefore, a protein concentration of 5 g per portion would be enough to comply with the FDA fortification requirements. A traditional corn tortilla in Mexico is 30 g on average. Considering a traditionally commercial-size tortilla (30 g), a fortification with a CPH of 5% and a portion size of two pieces of tortillas would represent an average consumption of 4.75 g of soluble protein. These results were expected due to an increase in the percentage of the hydrolysate added. Increases in the solubility of hydrolyzed proteins have been reported [48,49], although there are not enough studies that report the impact of the application of plant protein hydrolysates in food products.

Moisture content in tortillas has been previously reported to be 40–50% [50,51,52]. It has been correlated with the increase of firmness (penetration force) in the staling process, along with the recrystallization of starch chains [52]. In the control tortillas, although there was an increase in moisture content observed, the penetration force increased, confirming the changes in texture on the tortilla after 7 days that was observed in other studies [50]. These changes are also attributed to starch retrogradation and syneresis [50,53]. For the fortified tortillas, the observed constant moisture over time can be explained due to the water holding capacity of proteins. Water holding capacity refers to the total amount of water that proteins can bind, and it is a physical property linked to the ability of a food structure to prevent water from being released from the three-dimensional structure of the protein [54,55,56]. Due to the different ways in which proteins interact with water, the effect of constant moisture in fortified tortillas can be explained by the changes in functionality of hydrolysates in regard to water holding capacity and higher water absorption capacity [57,58]. Depending on the structure of the protein, a water molecule can interact with the proteins, adding polar groups on the protein surface through hydrogen bonds, through electrostatic interactions with amino acid sidechains, or through hydrophobic interactions [56]. In this case, the characteristics of the chickpea hydrolysate (high hydrophobicity, size, and charge of the peptides) are expected to facilitate interactions between molecules of water and hydrophilic groups of the protein side chains via hydrogen bonding, and therefore the increased water holding capacity [55,56]. Thus, the addition of hydrolysates as ingredients can increase water holding capacity [59,60,61], showing no difference in moisture over time.

Previously, it has been found that texture in maize tortillas can vary due to differences related to the grain since different maize varieties provide different rheological properties to the final product [41]. For a maize tortilla to be strong and flexible, protein–protein and protein–starch interactions are key [62,63]. The addition of extra protein in the form of hydrolysates affects these interactions, impacting on the texture, as reported before [20,43,62,63]. As observed in the results, the texture of a fresh white tortilla was impacted at higher fortification levels (10% and 15%), and the texture after 7 days remained with no difference, demonstrating that higher fortification levels can help in keeping the texture constant over time. The results for fresh blue maize tortillas showed that a higher amount of hydrolysate (15%) decreased the hardness of the tortilla. Some studies have shown that the interaction of phenolic compounds with proteins affects not only the water binding capacity but also the protein–protein interactions, and therefore the texture [59]. These interactions depend on the structure of the phenolic compounds and the protein, as well as on the protein concentration. In this case, we observed that at 15% fortification level, phenolic compounds seem to increase the interaction with the proteins, reducing the protein–water interactions and having an impact on the lower hardness value.

Changes in texture reported in previous fortification studies [64] of tortillas using pulses showed that the use of a protein concentrate of *Phaseolus lunatus* beans at 7.5% and added lysine (0.15%) and tryptophan (0.3%) had an impact on cohesion, reducing this texture parameter. This is in comparison with the present study, where the use of a chickpea hydrolysate at 15% or lower did not affect the cohesion of the tortillas tested. Additionally, Lecuona et al. [64] also reported changes in hardness at a 7.5% fortification of bean protein concentrate, enough to show increase in hardness in white maize tortillas. According to the results reported in this study, it is with fortifications of 10% and higher that the hydrolysate had an impact on the hardness of white maize tortillas.

As observed in the chickpea hydrolysate composite sample, the DPPIV inhibitory activity obtained by using bromelain to hydrolyze chickpea protein isolates is higher than the bioactivity that has been previously found in pepsin–pancreatin digests [31].

After the application of the hydrolysate in the maize tortilla, a higher bioactivity was shown in blue tortillas. In blue maize, the main phenolic compound that has been previously associated with maize tortillas is ferulic acid. Ferulic acid has shown antioxidant, anti-inflammatory, and anticarcinogenic properties [58,65,66]. Other phenolics, anthocyanins, have also been studied and have shown potential to modulate protein activity in pathways involved in insulin secretion, insulin resistance, and carbohydrate absorption [67]. The interaction between phenolic compounds and enzymes can potentially destabilize the enzyme position or orientation of substrate binding or catalytic residues, inhibiting the enzyme activity and increasing DPPIV inhibition [68]. Thus, phenolic compounds present in blue maize tortillas can potentially inhibit DPPIV, explaining the higher bioactivity shown from 0% to 10% fortification ratios. However, at 15% fortification, both blue and white tortillas showed no differences in their DPPIV inhibitory activity. This can be due to the higher protein concentration, since some studies have analyzed the interaction of phenolic compounds and proteins and have observed that in the presence of high protein concentrations, polyphenols interact at the hydrophobic sites of proteins, which might also influence bioactivity [59].

Studies on bioactivity after gastrointestinal digestion have shown different results due to the complexity of the process, and wide differences have been reported from in vitro and in vivo studies. In general, the stability of bioactive compounds through digestion seems to be related to the composition and length of the compound tested [69]. Further analyses are needed to understand how the digestive process can change the bioactivity of chickpea hydrolysates in animal or clinical studies.

Further statistical correlations showed a negative correlation between hue and DPPIV activity (Table S1); the higher the hue value, the lower the DPPIV inhibition.

The relevance of this research is the application of a chickpea hydrolysate with DPPIV inhibition that not only improved the soluble protein concentration of the tested product but also kept its bioactivity after processing conditions. In comparison with protein concentrates or isolates that have been used in the past to fortify food products, the optimized chickpea hydrolysate showed very good solubility and good physical characteristics that have the potential to be accepted by consumers.

## 5. Conclusions

Chickpea optimized bromelain hydrolysate produced from chickpea isolated proteins showed a high DPPIV inhibitory capacity. There was an increased DPPIV inhibitory activity as the fortification ratio increased. Tortilla fortification at 5% (*w*/*w*) level of hydrolysate led to a positive increase in soluble protein of 105% compared with the control tortilla. The bioactivity remained after processing conditions of dry cooking for 2 min at 232 °C during tortilla preparation. Nevertheless, fortification resulted in changes in color and texture that can have an impact on consumer acceptability. The optimized CPH has the potential to be used in a different range of food categories to improve soluble protein concentration and bioactivity to prevent T2DM and benefit its management. Further research is needed to evaluate the sensory characteristics, consumer acceptance, and clinical effect of CPH on DM indicators.

## Figures and Tables

**Table 1:** Peptides sequences determined by LC-ESI-MSMS from optimized chickpea hydrolysate with in silico potential DPPIV inhibitory activity obtained from isolated protein using bromelain ^+^.

Peptide Sequence	Molecular Mass (g/mol)	Blast Match Parental Protein	PI	Net Charge	Hydrophobicity (Kcal * mol ^−1^)	Chemical Structure
KDGGTAPAAGSGGGGAR	1385.663	Metabolic protein	9.8	1	27.05	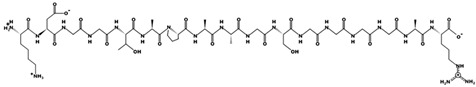
LVSGGGGAR	772.418	Metabolic protein	11.11	1	13.56	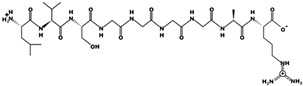
FDLPAL	674.363	2S Albumin-like, 11S Globulin seed storage protein 2-like	3.12	−1	7.97	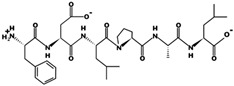
GPGGGGR	556.271	Trypsin inhibitor	11.13	1	15.60	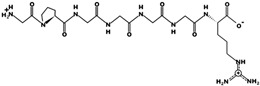
SPGAGAP	555.265	Metabolic protein	5.18	0	11.94	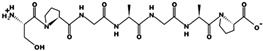
HTGAGV	540.265	Metabolic protein	7.69	0	12.82	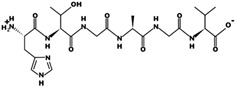
FGAR	449.238	Metabolic protein	10.9	1	9.65	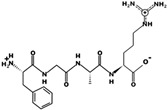
LPGR	441.269	Metabolic protein	11.11	1	9.75	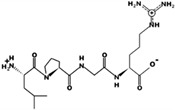
LVGGP	441.258	Metabolic protein	5.23	0	8.63	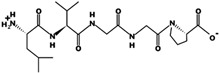
VAGR	401.238	Metabolic protein	11.11	1	10.90	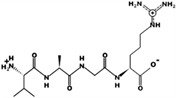
LLR	400.279	Metabolic protein	11.11	1	7.21	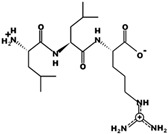

^+^ Peptides obtained from LC-ESI-MSMS elution profile with intensity of at least 50%; sequences from *Cicer arietinum* proteins were compared with BLAST tool.

**Table 2:** Soluble protein and color changes of tortillas fortified with different ratios of optimized chickpea hydrolysate with DPPIV inhibitory activity obtained from isolated protein using bromelain.

Ratios of Chickpea Hydrolysate Added to Tortilla (%, *w*/*w*)		Soluble Protein (g/100 g Tortilla)	L*	a*	b*	Hue Angle (Radians)	ΔE	Color Match
White maize tortilla	0	4.02 ± 0.91 d	73.47 ± 0.67 a	2.00 ± 0.01 c	26.90 ± 0.01 c	1.50 ± 0.01 a	n/a	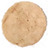
	5	7.65 ± 0.80 c	71.78 ± 0.04 a	2.56 ± 0.02 c	28.71 ± 0.04 bc	1.48 ± 0.01 b	3.10 ± 0.01	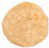
	10	10.46 ± 0.43 b	73.08 ± 1.16 a	3.70 ± 0.64 b	30.74 ± 2.86 b	1.45 ± 0.01 c	4.42 ± 3.01	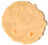
	15	13.06 ± 1.12 a	69.07 ± 0.01 b	5.30 ± 0.01 a	34.56 ± 0.03 a	1.42 ± 0.01 d	9.81 ± 0.02	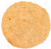
Blue maize tortilla	0	4.03 ± 0.04 d	29.85 ± 0.11 d	−0.64 ± 0.07 d	2.89 ± 0.10 c	1.78 ± 0.01 a	n/a	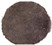
	5	8.21 ± 0.70 bc	42.57 ± 0.35 b	5.12 ± 0.12 c	6.46 ± 0.12 b	0.90 ± 0.01 b	14.08 ± 0.05	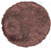
	10	10.05 ± 0.24 b	38.71 ± 0.14 c	7.07 ± 0.25 b	6.49 ± 0.37 b	0.74 ± 0.05 d	11.86 ± 0.04	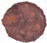
	15	12.61 ± 1.76 a	45.86 ± 0.17 a	8.50 ± 0.36 a	9.09 ± 0.06 a	0.82 ± 0.01 c	19.04 ± 0.03	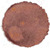

Results show the mean soluble protein concentration and color values ± standard deviation. Different letters indicate significant differences (*p* < 0.05) among fortification treatments. L* = lightness value. a* = axis is relative to green–red opponent colors (negative values = green, positive values = red). b* axis represents blue–yellow opponents (negative numbers = blue, positive = yellow).

**Table 3:** Thickness and diameter of fresh tortillas and moisture over storage time of tortillas fortified at different ratios with optimized chickpea hydrolysate with DPPIV inhibitory activity obtained from isolated protein using bromelain.

Fortification Ratio (%, *w*/*w*)		Thickness (mm)	Diameter (mm)	Moisture (%)		
		Fresh Tortilla	Fresh Tortilla	0 h	48 h	7 days
White maize tortilla	0	2.09 ± 0.25 a	80.08 ± 1.33a	34.47 ± 1.65 Bc	70.97 ± 7.30 Aa	64.93 ± 4.77 Aa
	5	1.80 ± 0.09 a	81.13 ± 1.23 a	43.86 ± 0.40 Ab	52.04 ± 6.36 Abc	45.64 ± 5.98 Ab
	10	2.03 ± 0.06 a	82.91 ± 0.19 a	52.09 ± 4.84 Aa	55.24 ± 8.49 Aab	59.87 ± 3.91 Aa
	15	1.87 ± 0.12 a	80.11 ± 1.14 a	36.94 ± 1.81 Abc	37.28 ± 0.31 Ac	41.25 ± 3.21 Ab
Blue maize tortilla	0	2.09 ± 0.10 a	81.89 ± 0.33 a	39.48 ± 4.12 Bb	56.75 ± 8.94 Aa	48.23 ± 2.87 ABab
	5	1.94 ± 0.10 a	81.07 ± 1.27 a	46.29 ± 4.31 Ab	38.19 ± 8.01 Aa	44.83 ± 2.29 Ab
	10	2.17 ± 0.09 a	79.11 ± 1.09 ab	59.94 ± 3.81 Aa	55.45 ± 9.85 Aa	56.28 ± 299 Aa
	15	1.97 ± 0.13 a	78.00 ± 0.94 b	36.55 ± 2.82 Ab	36.08 ± 4.92 Aa	37.91 ± 4.50 Ab

Results show the mean of thickness, diameter, and moisture percentage ± standard deviation. Different letters among samples indicate significant differences (*p* < 0.05). Lowercase shows significant difference among fortification treatments. Capital letters show significant difference among storage times. Statistical analysis was made separately for white and blue maize tortillas.

**Table 4:** Texture analysis of tortillas fortified with optimized chickpea hydrolysate with DPPIV inhibitory activity obtained from isolated protein using bromelain at different ratios and at different storage times.

Fortified Tortilla (% *w/w*)		0 h			48 h			7 days		
		Hardness (N)	Cohesiveness	Penetration Force (N)	Hardness (N)	Cohesiveness	Penetration Force (N)	Hardness (N)	Cohesiveness	Penetration Force (N)
White maize tortilla	0	13.56 ± 1.66 Bb	0.90 ± 0.04 Aa	0.18 ± 0.01 Ba	16.97 ± 0.49 Ab	0.92 ± 0.05 Aa	0.18 ± 0.02 Bb	3.63 ± 0.37 Cd	0.91 ± 0.02 Aa	0.35 ± 0.04 Aa
	5	9.96 ± 0.76 Bb	0.89 ± 0.03 Aa	0.22 ± 0.05 Aa	12.15 ± 3.15 Bb	0.92 ± 0.05 Aa	0.26 ± 0.05 Aa	18.60 ± 0.48 Ac	0.94 ± 0.03 Aa	0.35 ± 0.10 Aa
	10	27.47 ± 3.18 Ba	0.94 ± 0.02 Aa	0.15 ± 0.04 Aa	25.51 ± 1.96 Ba	0.93 ± 0.05 Aa	0.20 ± 0.01 Aa	32.38 ± 1.12 Aa	0.95 ± 0.03 Aa	0.20 ± 0.03 Ab
	15	25.58 ± 4.01 Aa	0.90 ± 0.04 Aa	0.21 ± 0.01 Aa	24.24 ± 2.02 Aa	0.90 ± 0.05 Aa	0.21 ± 0.03 Aa	25.39 ± 1.51 Ab	0.96 ± 0.01 Aa	0.21 ± 0.03 Aab
Blue maize tortilla	0	26.63 ± 1.99 Aa	0.95 ± 0.02 Aa	0.13 ± 0.01 Cc	14.34 ± 0.95 Ba	0.93 ± 0.04 Aa	0.20 ± 0.02 Bb	5.74 ± 0.35 Cc	0.91 ± 0.05 Aa	0.61 ± 0.03 Aa
	5	20.34 ± 3.97 Aa	0.89 ± 0.03 Aa	0.28 ± 0.02 Ba	19.50 ± 3.72 Aa	0.92 ± 0.04 Aa	0.28 ± 0.03 Ba	11.43 ± 0.48 Bb	0.94 ± 0.01 Aa	0.34 ± 0.01 Ab
	10	26.39 ± 3.76 Aa	0.94 ± 0.03 Aa	0.13 ± 0.02 Bc	14.38 ± 2.55 Ba	0.91 ± 0.05 Aa	0.20 ± 0.01 Ab	21.53 ± 2.45 Aa	0.94 ± 0.01 Aa	0.18 ± 0.03 Ac
	15	16.71 ± 2.63 Ab	0.91 ± 0.02 Aa	0.22 ± 0.02 Bb	12.98 ± 2.84 Aa	0.93 ± 0.02 Aa	0.25 ± 0.01 ABa	13.38 ± 0.90 Ab	0.93 ± 0.03 Aa	0.29 ± 0.03 Ab

Results show the mean texture values ± standard deviation. Different letters among samples indicate significant differences (*p* < 0.05). Lowercase shows significant difference between fortification treatments. Capital letters show significant difference among store time.

## Data Availability

The data are contained within the article. The data presented in this study are available in the present article.

## Supplementary Materials

The following are available online at https://www.mdpi.com/article/10.3390/foods10081835/s1, Table S1: Correlations among different parameters of blue and white fortified tortillas, Figure S1: Mass spectra indicating elution time, peptide mass, and precursor ion *M/z* value of peptide GPGGGGR, Figure S2: Low molecular weight protein profile of blue (B) and white (W) maize tortillas fortified with different chickpea hydrolysate ratios (0%, 5%, 10%, 15%), Figure S3: TPA graphics of white tortilla fresh and after 7 days (control and 15% fortification). (a) TPA fresh control white tortilla, (b) TPA control white tortilla after 7 days, (c) TPA fresh white tortilla with 15% fortification level, (d) TPA white tortilla with 15% fortification level after 7 days.
