# Supplementary material for: Fortification of Maize Tortilla with an Optimized Chickpea Hydrolysate and Its Effect on DPPIV Inhibition Capacity and Physicochemical Characteristics
Source: Foods. 2021 Aug 9;10(8):1835. doi: 10.3390/foods10081835 (PMC8392616; doi:10.3390/foods10081835)
Supplement: Supplementary file 1 [file foods-10-01835-s001.zip › foods-1296076-supplementary.pdf]

Supplemental Table S1. Correlations among different parameters of blue and white fortified tortillas.

**Blue maize tortillas correlations**				
DPPIV inhibition	Hue	Hardness (fresh)	Moisture (fresh)	Puncture force (fresh)
DPPIV inhibition	**0.015**	0.373	0.700	0.482
Hue	**0.015**	0.506	0.544	0.557
Hardness (fresh)	0.373	0.506	0.461	0.172
Moisture (fresh)	0.700	0.544	0.461	0.665
Puncture force (fresh)	0.481	0.557	0.172	0.665
**White maize tortillas correlations**				
DPPIV inhibition	Hue	Hardness (fresh)	Moisture (fresh)	Puncture force (fresh)
DPPIV inhibition	0.151	0.420	0.468	0.856
Hue	0.151	0.180	0.806	0.916
Hardness (fresh)	0.420	0.180	0.629	0.518
Moisture (fresh)	0.468	0.806	0.629	0.428
Puncture force (fresh)	0.856	0.916	0.518	0.428

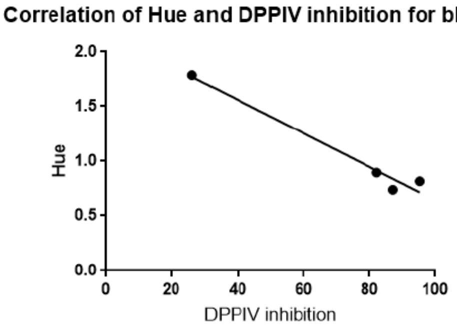

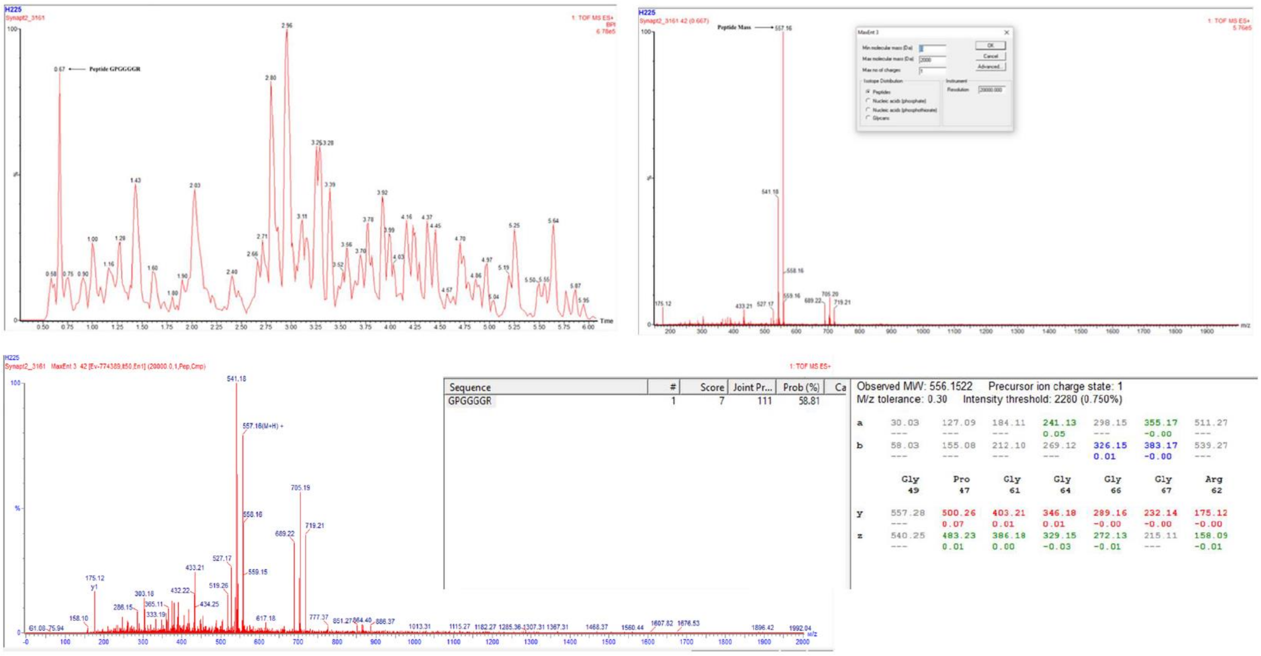

Figure S1. Mass spectra indicating elation time, peptide mass and precursor ion M/z value of peptide GPGGGGR.

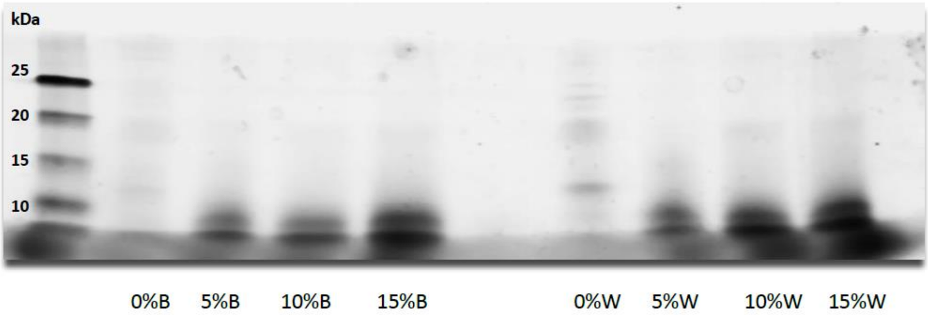

Figure S2. Low molecular weight protein profile of blue (B) and white (W) maize tortillas fortified with different chickpea hydrolysate ratios (0%, 5%, 10%, 15%).

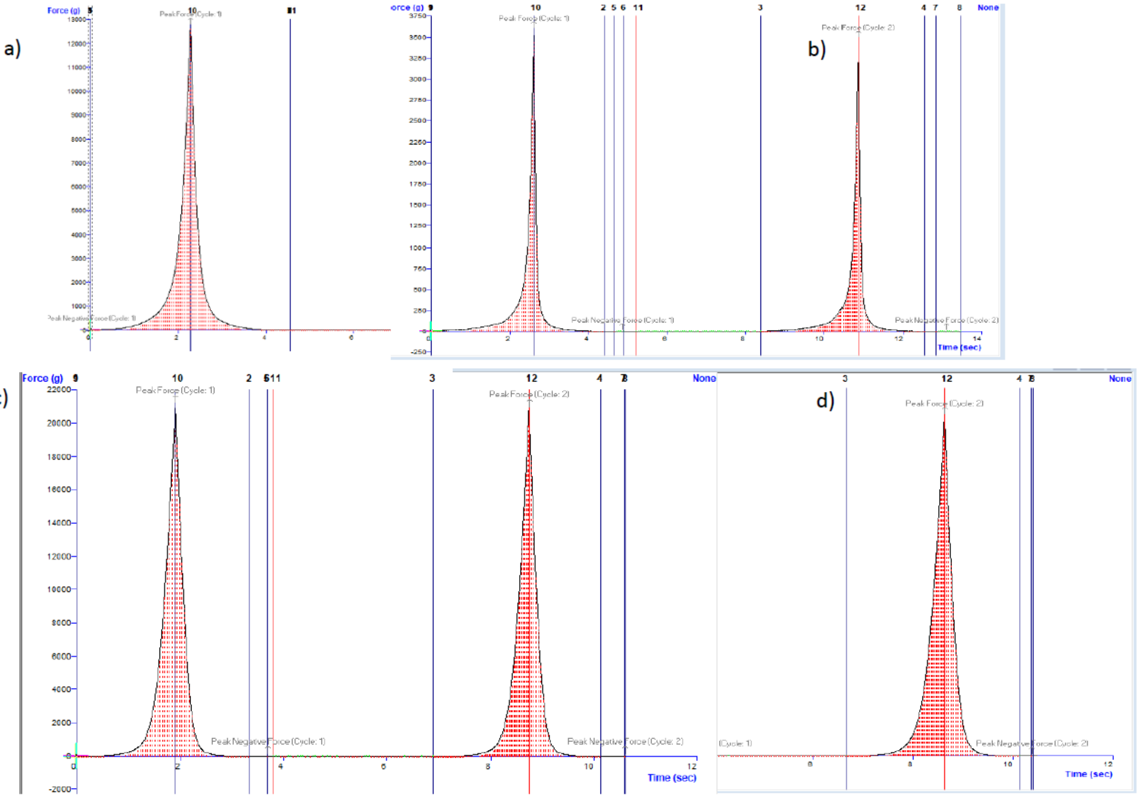

Figure S3. TPA graphics of white tortilla fresh and after 7 days (control and 15% fortification). a) TPA fresh control white tortilla, b) TPA control white tortilla after 7 days, c) TPA fresh white tortilla with 15% fortification level, d) TPA white tortilla with 15% fortification level after 7 days.
